# Rapid promoter evolution of male accessory gland genes is accompanied by divergent expression in closely related *Drosophila* species

**DOI:** 10.1093/genetics/iyaf226

**Published:** 2025-10-18

**Authors:** David W J McQuarrie, Frannie H S Stephens, Alexander D Ferguson, Roland Arnold, Alberto Civetta, Matthias Soller

**Affiliations:** School of Biosciences, College of Life and Environmental Sciences, University of Birmingham, Edgbaston, Birmingham B15 2TT, United Kingdom; School of Biosciences, College of Life and Environmental Sciences, University of Birmingham, Edgbaston, Birmingham B15 2TT, United Kingdom; School of Biosciences, College of Life and Environmental Sciences, University of Birmingham, Edgbaston, Birmingham B15 2TT, United Kingdom; Department of Cancer and Genomic Sciences, College of Medicine and Health, University of Birmingham, Birmingham, B15 2TT, United Kingdom; Department of Biology, University of Winnipeg, Winnipeg, Canada MB R3B 2E9; School of Biosciences, College of Life and Environmental Sciences, University of Birmingham, Edgbaston, Birmingham B15 2TT, United Kingdom; Division of Molecular and Cellular Function, School of Biological Sciences, University of Manchester, Oxford Road, Manchester M13 9PT, United Kingdom

**Keywords:** accessory gland proteins, seminal fluid proteins, promoter evolution, evolution hot-spot, hybrid dysgenesis, post-mating response

## Abstract

Seminal fluid proteins (Sfps) are essential for reproductive success and evolve fast on average, possibly driven by post-copulatory sexual selection (PCSS) originating from sperm competition and cryptic female choice. Counterintuitively, however, the coding region only in few Sfps evolves adaptively. Hence, additional genomic and functional factors must play a role in Sfp evolution independent of the protein coding region. To shed light on drivers of Sfp evolution we focus on those Sfps predominantly expressed in male accessory glands of *Drosophila* to examine their evolution in the tissue which produces the majority of Sfps. Unlike the testis, the accessory glands are known to develop normally in hybrids, allowing us to control for cellular environment differences arising during speciation. Here, we identify hotspots of rapid evolution in accessory gland protein genes (Acp) promoters, driven by base changes and insertions/deletions (indels). We further show that changes in promoter sequences are accompanied by gene expression divergence among closely related species. Using hybrids, we demonstrate that species-specific expression divergence is maintained for some Acps, while others exhibit dominance of one allele. These results indicate that regulatory evolution, rather than genome background variation, drives Acp expression changes and promotes their rapid evolution.

## Introduction

During copulation, sperm together with seminal fluid is transferred from males to females. Seminal fluid proteins (Sfps) have a range of vital functions, including regulation of sperm storage, sperm survival and fertilization, but also include complex roles in sperm competition (Ravi Ram and Wolfner 2007; Avila et al. 2010, 2011; Sirot et al. 2014; Civetta and Ranz 2019; Schjenken and Robertson 2020; Wigby et al. 2020; Wainwright et al. 2021). Moreover, Sfps trigger a wide range of physiological and behavioral changes in females after mating that contribute to reproductive success (Kubli 2003; Avila et al. 2011). Most Sfps are products of the male reproductive tissue the accessory glands, called accessory gland proteins (Acps) (Wolfner 1997). A key regulator of the female post-mating response in *Drosophila melanogaster* is sex-peptide (SP), that upon transfer during mating enters the hemolymph and passes the blood–brain barrier to target command neurons in the brain that reduce remating and induce oviposition (Haussmann et al. 2013; Nallasivan et al. 2024). In addition, SP regulates many other aspects of the female post-mating response including increased egg production, feeding, changes in food choice, sleep, memory, constipation, midgut morphology, and stimulation of the immune system (Soller et al. 1999; Peng et al. 2005; Carvalho et al. 2006; Domanitskaya et al. 2007; Isaac et al. 2010; Kim et al. 2010; Ribeiro and Dickson 2010; Cognigni et al. 2011; Garbe et al. 2016; Scheunemann et al. 2019; White et al. 2021).

In general, reproductive genes evolve more rapidly than non-reproductive genes. Sfps are some of the fastest-evolving genes on average, with some lacking detectable similarities or orthologs between closely related species (Swanson et al. 2001; Swanson and Vacquier 2002; Avila et al. 2011; Rowe et al. 2019; Sirot 2019; Wigby et al. 2020, 2022; Patlar et al. 2021; Hurtado et al. 2022b; Ranz et al. 2025). Fast evolution of Sfps has to a large degree been attributed to post-copulatory selection (PCSS) originating from sperm competition and cryptic female choice due to widespread polyandry (Boorman and Parker 1976; Clark et al. 1999; Birkhead and Pizzari 2002; Ravi Ram and Wolfner 2007; Wigby et al. 2020). Surprisingly, however, coding regions of only a few Sfp genes are under positive selection in *Drosophila* (Aguadé et al. 1992; Aguadé 1999; Begun et al. 2000; Holloway and Begun 2004; Andrés et al. 2006; Haerty et al. 2007; Rowe et al. 2019). Rapid coding sequence evolution of most Sfps between *Drosophila melanogaster* and *D. simulans* has been shown to occur through relaxed selection, allowing increased genetic variation within and between species (Patlar et al. 2021).

Of note, expression of Sfp genes rapidly diverges between related species (Coulthart and Singh 1988; Ellegren and Parsch 2007; Harrison et al. 2015; Kratochwil et al. 2018; Cridland et al. 2020; Flacchi et al. 2024). These observations support that divergence in gene expression between species can lead to phenotypic changes among species without altering the amino acid sequence (Kratochwil et al. 2018; Hagen et al. 2019; Caglayan et al. 2023). Consequently, these expression changes in Sfp genes could influence female post-mating fitness and be selectively favored for driving adaptive processes. In fact, a recent survey of selection acting upon expression of Sfp genes uncovered enrichment for both stabilizing and directional selection for Sfps with reproductive-tissue-specific expression (Flacchi et al. 2024). Although promoter sequence changes have been shown to correlate with expression divergence (Wray 2007; Wittkopp and Kalay 2011), how such changes are implemented to produce species-specific adaptations remains poorly understood.

One possible mechanism driving expression changes is the mutagenic impact of transposon insertions into promoter regions, which are particularly susceptible due to their open chromatin state. Such insertions can lead to drastic changes in gene expression, especially at the onset of speciation, and species-specific adaptations may cause hybrid incompatibilities, often resulting in sterility or lethality in hybrids of closely related species (Brideau et al. 2006). Promoters of germline genes, including piRNA pathway factors, are particularly relevant in this context because piRNAs suppress transposon mobilization (Czech and Hannon 2016; Yamashiro and Siomi 2018). These promoters evolve rapidly, suggesting that regulation of transposon silencing has a strong impact on promoter evolution (McQuarrie et al. 2024). A striking example comes from nuclear pore complex (NPC) genes, which mediate selective transport between the nucleus and cytoplasm and have also been implicated in piRNA-driven transposon silencing (Munafò et al. 2021). NPC genes play an unexpected role in speciation (Presgraves et al. 2003; Tang and Presgraves 2009; Presgraves 2010), and a promoter deletion allele in the NPC gene *Nup54* causes SP insensitivity through defects in neuronal wiring (Nallasivan et al. 2021). Since the *Nup54* promoter accumulates indels and base changes rapidly, regulatory evolution is likely to contribute to speciation through sexual conflict (Nallasivan et al. 2021). Systematic analysis has further shown that rapid evolution is a general feature of NPC genes (McQuarrie et al. 2023). This highlights how promoter evolution in one class of genes can provide a model for understanding regulatory divergence more broadly, including in other rapidly evolving gene groups.

To specifically address whether rapid evolution of promoters is a key feature in the process of speciation, we focused on genes coding for Acps because they constitute the majority of Sfps, are expressed in a single tissue consisting of only two main cell types and expression of these genes can be analysed in bulk RNA-seq datasets of closely related species and hybrids (Bertram et al. 1992; Wolfner 1997). Our analysis reveals that promoters of Acps evolve fast, and that their coding regions also rapidly accumulate sequence changes (base changes or indels) between species. Moreover, these changes in promoters are paralleled by changes in expression between closely related species. While some Acp expression changes are maintained in hybrids, others show dominance for one species. These findings suggest that promoter changes, rather than genomic background, play a significant role in driving expression changes, indicating that promoter evolution may contribute to the rise of new species.

## Materials and methods

### Defining Acp genes

We based our list of Acp genes on previous published lists (Wigby et al. 2020; Hurtado et al. 2022a, 2022b), and restricted our selection to genes with graded tissue specificity index τ ≥ 0.9, which was calculated for all genes from FlyAtlas2 RNA expression data (Yanai et al. 2004; Leader et al. 2017). Acp genes with τ ≥ 0.9 are genes with highly concentrated expression in the accessory glands, and very low expression elsewhere. A total of 155 Acps from the original list met these criteria (Supplementary Dataset 1). As a control group, a random sample of 155 genes taken from the genome, excluding the 155 Acps, was defined. Randomization was performed using the sample function in base R (R Core Team 2022). As a further control group, we analysed the top 50 genes with highly concentrated expression in the Malpighian tubules (τ ≥ 0.9).

### Sequence/data retrieval and alignment

Genomic sequences, PhyloP27way data and CAGE data were retrieved from UCSC Genome Browser using the UCSC Table Browser sequence retrieval tool (Kent et al. 2002; Karolchik 2004). A standardized sequence region of 2000 bases upstream of the annotated gene TSSs was exported for each gene to ensure inclusion of promoter regions. A region of 1000 nucleotides upstream and 300 nucleotides downstream of gene TSSs was collected from PhyloP27way data. Where genes had multiple TSSs, dominant transcripts were identified using available CAGE values proximal to annotated TSSs. To identify dominant TSSs while accounting for CAGE peak position inaccuracy, a nine-nucleotide sliding window score (*f*) was calculated using R version 4.4.2 for full gene lengths (R Core Team 2022). Annotated TSSs with the highest *f* score per gene were considered dominant and used as representative transcripts for analysis. Sequences were aligned with clustalW using the R package msa and manually refined using the MEGA11 package (Bodenhofer et al. 2015; Tamura et al. 2021).

### Identification of promotor hotspots and comparison of substitution rates

To analyse promoter evolution hotspots, gene and promoter sequences were adjusted to span −1000/+300 nucleotides from the TSS of each gene. To assess the occurrence of sequence change hotspots within promoter sequences, we computed the hotspot accumulation score (*d*) using alignments derived from the trimming process, or alternatively, PhyloP data. These alignments were converted into “events” for each species when compared to *D. melanogaster*. Here, each nucleotide in the sequence was assigned a value of 0 if it represented a conserved sequence and 1 if it indicated a sequence change (either a base change or an indel event). Events were tallied for all changes, as well as separately for base changes and indels. The cumulative events were calculated for both concatenated gene groups and individual genes across all nucleotides. Subsequently, a sliding event (*Se*) score was computed from these tallies using a sliding window of five bases along the sequence. To determine the percentage of events surpassing the average control promoter *Se* score (*d*), a 350-nucleotide region upstream of the estimated TATA box region was examined. This involved dividing the total number of *Se* scores exceeding the average sliding event score of the control group (*Se^C^*) by the total number of events within that region (*N*). To compute the promoter region scores (*d^P^*) from PhyloP data, we substituted the total number of PhyloP (*p*) scores in the 350-nucleotide regions that were below the average of the control group promoter region (*p^C^*) in place of the total number of *Se* events where *Se* was greater than *Se^C^* as previously described. Significance was assessed using non-parametric chi-squared tests compared to the control group *d* score. Significance values where *P* ≤ 0.05 after Bonferroni correction were considered statistically significant.

### Accumulation of substitutions along extended gene regions

To test for nonrandom accumulation of indels along the Acp extended gene regions between the five analysed *Drosophila* species (*D. melanogaster*, *D. simulans*, *D. sechellia*, *D. yakuba*, and *D. erecta*) we determined significant deviations from a uniform distribution of substitutions using an empirical cumulative distribution function (Civetta et al. 2016). The position of the indel event was defined as the 5′ site of the start of the indel in the alignment (Schaeffer 2002). The function (G) detects monotonic increases in substitutions (n) measured as the difference between the relative occurrence of a nucleotide change and its relative position in the alignment (Civetta et al. 2016). Whether differences between the values of the G function (ΔG) between substitutional events deviate from a random accumulation of changes is tested using Monte Carlo simulations to produce 100,000 samples of n events by sampling sites without replacement along the alignment (Civetta et al. 2016).

### Comparative gene expression analysis between *D. melanogaster*, *D. simulans* and *D. yakuba*

We utilized publicly available RNA-seq data from *D. melanogaster*, *D. simulans*, and *D. yakuba* male whole flies (GSE28078) and from *D. melanogaster* and *D. simulans* hybrid male accessory glands (PRJNA913156) for our analyses (Graveley et al. 2011; Majane et al. 2024). Adapter sequences were removed from the raw reads using the Trimmomatic tool (v0.39) with default parameters (Bolger et al. 2014). Trimmed reads were aligned to the respective genome assemblies (dm6 for *D. melanogaster*, Prin_Dsim_3.1 for *D. simulans*, and droYak2 for *D. yakuba*) using the RNA-seq aligner RNA STAR (v2.7.10b) (Dobin et al. 2013). Gene counts were quantified using the featureCounts tool from the Subread package (v2.0.3) (Liao et al. 2013). Orthologous genes were identified using the Flybase ftp accession orthologue lookup table (Gramates et al. 2022). The initial analysis steps were performed using usegalaxy.eu (Community 2022). For the interspecies differential gene expression comparison, Transcript per Million (TPM) values were computed to mitigate potential influence of differences in gene lengths of orthologs in the different species. TPMs for orthologs in all three species were extracted using the TPMCalculator package (Vera Alvarez et al. 2019). Differential gene expression analysis was then performed using Limma and EdgeR packages implemented in iDEP (Ritchie et al. 2015; Ge et al. 2018; Chen et al. 2025). The same pipeline was applied to *D. melanogaster*/*D. simulans* hybrid expression data (*n* = 3) after aligning to a combined reference genome and then calling TPM for each species separately, resulting in three *D. melanogaster* and three *D. simulans* expression sets; reads with multiple alignments (indistinguishable between both species) and expression in less than 4 samples (avoidance of mono-allelic expression inherited from one species, i.e. the X chromosome genes inherited exclusively from the *D. simulans* parent) were removed.

### *Drosophila* stocks, genetics, and hybrid culture

Wild type *D. melanogaster* CantonS, *D. simulans* and *D. sechellia* Jallons (S-32) were used and kept on standard cornmeal-agar food (1% industrial-grade agar, 2.1% dried yeast, 8.6% dextrose, 9.7% cornmeal and 0.25% Nipagin, all in (w/v)) in a 12 h light:12 h dark cycle. To obtain hybrid males, *D. melanogaster* males were crossed to either *D. simulans* or *D. sechellia* virgin females.

### RNA extraction and RT-PCR

Total RNA extraction was carried out using Tri-reagent from SIGMA according to the manufacturer's instructions. Reverse transcription was performed using Superscript II (Invitrogen) using an oligo-dT17 V primer as previously described (Haussmann et al. 2011). PCR was performed for 40 cycles with 1 μl of cDNA. Primers for analysis of hybrid gene expression were CG30486_F1 (GTGTCTGCCGGAAATAACGCATAT), CG30486_R1 (TCCGTATTGCTGCAGTAATCGTC), Obp56f_F1 (TCTCTGCTATCTGGCTCCA), Obp56f_R1 (GAAGTCTAATGCTTGGTAATGGACT), CG11598_F1 (GTCTCTATATTGACCTTGCCAAGGGA), CG11598_R1 (GCCAATGAATCCTGAGGTCCGTTTAGCA), CG15117_F1 (TTGCGGACTTTAAGACAGCACAAA), CG15117_R1 (CGGTAAACTTATTTACAGTGGCCGAATA), Rpl32_F1ms (CCGCCACCAGTCGGATCGATATGCTAAG), Rpl32_R1ms+62 (GCTATCCCAATCTCAGAAATGACAATTGAACTCGGCACTCGCACATCATTTTTTAACTAAAAGTCCGGTATATTAACGTTTACAAATGTG). Restriction enzymes (AcuI, BsaWI, MluI, NgoMIV, Sau3AI, or ScaI) were from New England Biolabs and DNA restriction digests were performed according to manufacturer's instructions. Experiments included at least two biological replicates. PCR product bands were quantified using Quantity One 1-D Analysis Software 4.6.8 (BioRad) according to the manufacturer's instructions. Band intensities were normalized to gene length for digested fragments. Hybrid-allele differential gene expression was calculated as the log_2_ fold change for *D. melanogaster* relative to either *D. simulans* or *D. sechellia*.

## Results

### Promoters of accessory gland genes are hotspots for accumulation of indels and base changes

**Fig. 1.**
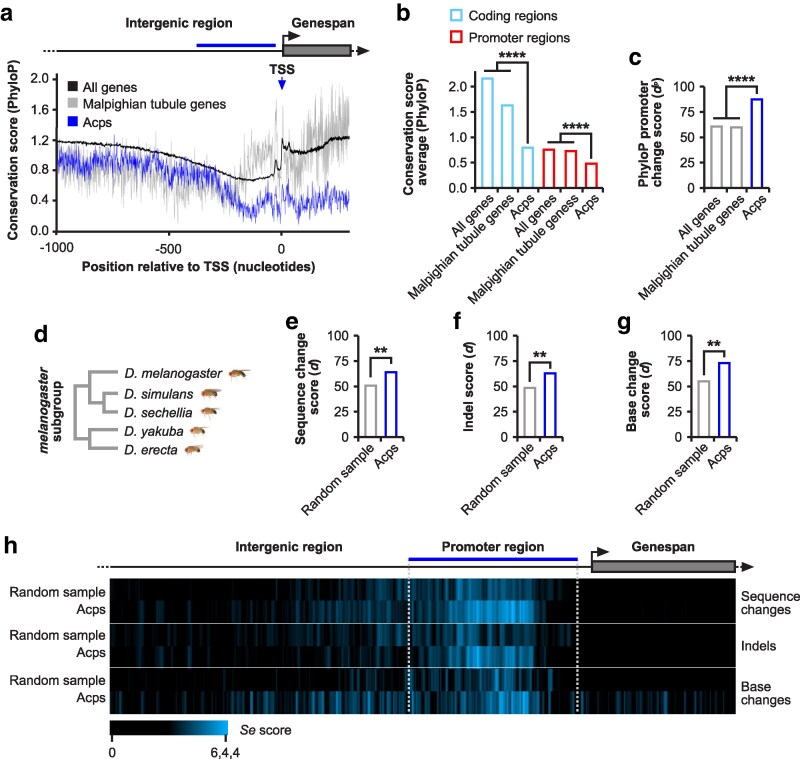
Promoters of accessory gland-specific Acp genes are hotspots for indel and base change accumulation. a) Metagene plot of average PhyloP27way conservation scores between Acps (blue), Malpighian tubule expressed genes (gray), and all other genes in the *Drosophila* genome (black). The blue line on the gene model indicates the analysed 350-nucleotide promoter region. b and c) PhyloP27way conservation score averages (b) and PhyloP27way promoter change *d^P^* scores (c) for the coding regions and/or the 350-nucleotide promoter regions of Acp genes compared to the Malpighian tubule expressed genes, and to all genes in the *Drosophila* genome. Statistically significant differences from unpaired Student t-tests (b) and non-parametric chi-squared tests (c) are indicated by asterisks (****P ≤ 0.0001). d) Phylogenetic tree of the *melanogaster* subgroup (*D. melanogaster*, *D. simulans*, *D. sechellia*, *D. yakuba*, and *D. erecta*) of *Drosophila* which were analysed in this study. *Drosophila* images were obtained from the D. J. Obbard Lab image collection (https://obbard.bio.ed.ac.uk/) under a CC BY-NC 4.0 licence. e–g) Sequence change quantification (*d*) for Acps (blue) compared to a random sample of genes (grey) between the *melanogaster* subgroup members (d). Statistically significant differences from non-parametric chi-squared tests are indicated by asterisks (**P ≤ 0.01). h) Heatmaps indicating a sliding window score of sequence change, indel, or base change accumulation −1000/+300 nucleotides from the TSS.

To analyse promoter evolution of Acps we first generated a list of 155 bona fide genes predominantly expressed in male accessory glands from annotated gene expression data (Yanai et al. 2004; Leader et al. 2017; Wigby et al. 2020; Hurtado et al. 2022a, 2022b) (Supplementary Dataset 1). Although some Acps show low-level expression outside the accessory glands (Thayer et al. 2024), we restricted our analysis to genes with a tissue-specificity index τ ≥ 0.9 in male accessory glands, a commonly used threshold for identifying tissue-specific genes (Yanai et al. 2004). We then used PhyloP27way data, a conservation score based on 27 insect species at each nucleotide position, to generate comparative metagene plots of regions 1000 nucleotides upstream and 300 nucleotides downstream of transcription start sites (TSS) of Acp genes and compared them to all other genes in the *D. melanogaster* genome (Fig. 1a). In addition, we analysed an additional control group comprised of genes near-exclusively expressed in the Malpighian tubules (τ ≥ 0.9), chosen as this tissue shares features with the accessory glands: both are adult secretory tissues that produce and release proteins, and both are primarily composed of two cell types (Dow et al. 2021). Quantification of the metagene plot values and analysis of mutation hotpots between the Acp genes, Malpighian tubule genes, and all non-Acp genes revealed that Acps accumulate mutations more rapidly in both their coding regions and promoters compared to the controls (Fig. 1, a to c). In addition, accumulation of mutations was also observed in 5` untranslated regions (UTRs) of Acps, in contrast to the 5` UTRs of NPC genes and piRNA factors (McQuarrie et al. 2023, 2024). Because Acp genes are generally small and compact genes, regulatory elements could possibly be in 5` UTRs (Supplementary Dataset 1, Fig. 1, a to c).

To understand whether promoter regions evolve rapidly between closely related *Drosophila* species, we focused on five species in the *melanogaster* subgroup (*D. melanogaster, D. simulans, D. sechellia, D. yakuba,* and *D. erecta*) (Fig. 1d). We quantified accumulation of sequence changes in promoters for insertions and deletions (indels), and base changes in Acps and compared them to an equal number of random sampled genes (Fig. 1, e to h). Our analysis confirmed that, compared to control genes, promoters of Acps evolve rapidly among closely related species by accumulating both indels and base changes.

### Rapidly diverging expression of Acp genes among closely related *Drosophila* species correlates with accumulation of mutations in promoters

**Fig. 2.**
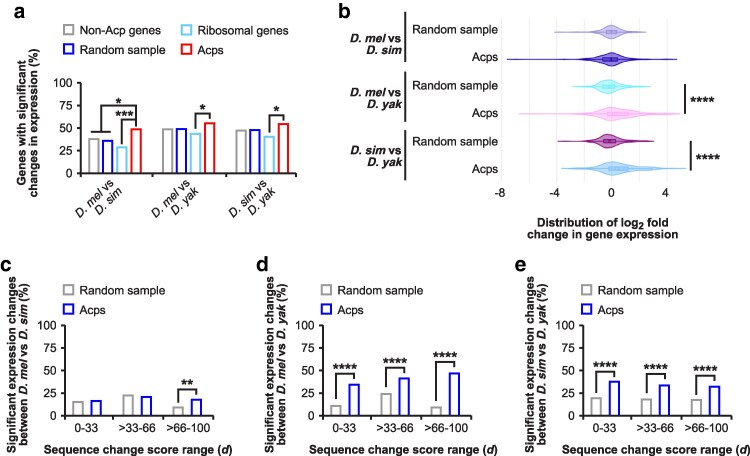
Expression of Acp genes diverges rapidly between closely related *Drosophila* species and correlates with mutation accumulation in promoters. a) The percentage of genes with significant changes in gene expression for Acps (red) compared to all analysed non-Acp genes (grey), a random sample of genes (blue), and ribosomal genes (light blue) based on differential gene expression analysis between *D. melanogaster* and *D. simulans*, *D. melanogaster* and *D. yakuba*, and *D. simulans* and *D. yakuba*. Statistically significant differences from FDR corrected non-parametric chi-squared tests are indicated by asterisks (*P ≤ 0.05, ***P ≤ 0.001). b) Violin plots of log_2_ fold changes in gene expression distribution for the random genome sample and Acps based on differential gene expression analysis between *D. melanogaster* and *D. simulans*, *D. melanogaster* and *D. yakuba*, and *D. simulans* and *D. yakuba*. Statistically significant differences from Mann–Whitney U tests are indicated by asterisks (****P ≤ 0.0001). c–e) Comparison of significant changes in log_2_ fold changes (>1 or <−1 log_2_ fold change) in gene expression in conservation promoter score (*d*) range 0 to 33%, >33 to 66%, and >66 to 100%. Expression changes for Acp (blue) and a random sample of genes were analysed between *D. melanogaster* and *D. simulans*, *D. melanogaster* and *D. yakuba*, and *D. simulans* and *D. yakuba*. Statistically significant differences from non-parametric chi-squared tests are indicated by asterisks (**P ≤ 0.01, ****P ≤ 0.0001).

To determine whether changes in promoter regions of Acp genes correlate with divergent gene expression, we examined differential gene expression in adult males between three pairs of closely related species in the *melanogaster* subgroup (*D. melanogaster* with *D. simulans*, *D. melanogaster* with *D. yakuba*, and *D. simulans* with *D. yakuba*). In this analysis we found that expression of Acp genes diverges more compared to all non-Acp genes and the random sample genes for the *D. melanogaster* vs *D. simulans* comparison (Fig. 2a). As a negative control we analysed highly conserved ribosomal component gene expression which showed significantly less divergence compared to the Acp genes for all comparisons (Fig. 2a). While the frequency of significantly diverging Acps is greatest between closely related species, the distribution of expression changes is more skewed in comparisons involving the more distantly related *D. yakuba* (Fig. 2b).

To analyse whether Acps with rapidly evolving promoters were also differentially expressed between species, we divided Acp and control genes into three conservation score groups based on the calculated *d* score (0 to 33, >33 to 66, >66 to 100 *d*) for each gene and plotted the percentage of genes with significant expression changes (>1 or <−1 log_2_ fold change) for each group (Fig. 2, c to e, Supplementary Dataset 1) (McQuarrie et al. 2023). The random sample control group generally displayed the highest differences in expression for the middle conservation score group (>33 to 66 *d*) (Fig. 2, c to e). However, Acps were enriched for significant expression changes in the faster evolving group (>66 to 100 *d*) across all species comparisons (Fig. 2, c to e). Of note, this trend was also observed in the other groups (0 to 33 and >33 to 66 *d*) for the species comparisons to *D. yakuba*, but not between *D. melanogaster* and *D. simulans* (Fig. 2, c to e).

### Acp gene promoters with divergent expression between species display positional hotspots for sequence changes

**Fig. 3.**
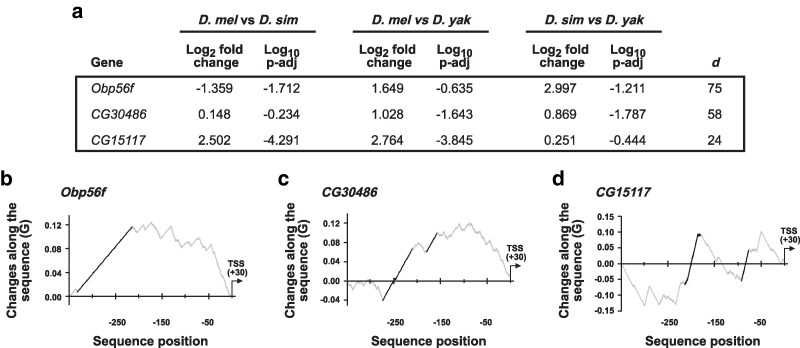
Acp gene promoters with divergent expression between species are hotspots for sequence change accumulation. a) Differential gene expression results for *Obp56f*, *CG30486*, and the control *CG15117* between *D. melanogaster* and *D. simulans*, *D. melanogaster* and *D. yakuba*, and *D simulans* and *D. yakuba*. Significance was considered where log_2_ fold change in gene expression was ≥0.5 or ≤−0.5, and the adjusted *P*-value was ≤0.05. b and c) Plots of G scores between nucleotide changes and the differential accumulation of events along fast evolving promoter sequences for *Obp56f* and *CG30486*. Sequences were aligned for *D. melanogaster*, *D. simulans*, *D. sechellia*, *D. yakuba*, and *D. erecta*. Positions in the alignments with significant stretches of substitutions (hotspots) are identified by black lines.

Next, we further molecularly analysed Acp genes with significant expression changes in promoter conservation in more detail. We selected three genes with consistent differences across pairwise species comparisons but with different levels of promoter evolution between *D. melanogaster*, *D. simulans*, and *D. yakuba*, (*Obp56f*, *CG30486*, and *CG15117*) (Fig. 3a, Supplementary Dataset 1). Analysis of sequence change hotspot accumulation in the promoters of these genes revealed substitution hotspots localized to promoters upstream of the TSSs for all three genes (Fig. 3, b to d).

### Divergent expression of Acps with fast evolving promoters is maintained in *Drosophila* hybrids

Interspecies comparisons of differential gene expression cannot rule out the effect of differences in the genomic background or resulting changes to the cellular environment that might have arisen during speciation. To test whether changes in the cellular environment of Acp genes can alter expression, we generated hybrids, with a mixed cellular environment, between *D. melanogaster* and *D. simulans* and between *D. melanogaster* and *D. sechellia.* In these hybrids, accessory glands are known to develop normally in contrast to the gonads (Wong et al. 2010), thus ruling out possibly allometric effects due to tissue atrophy (Sztepanacz and Houle 2021).

**Fig. 4.**
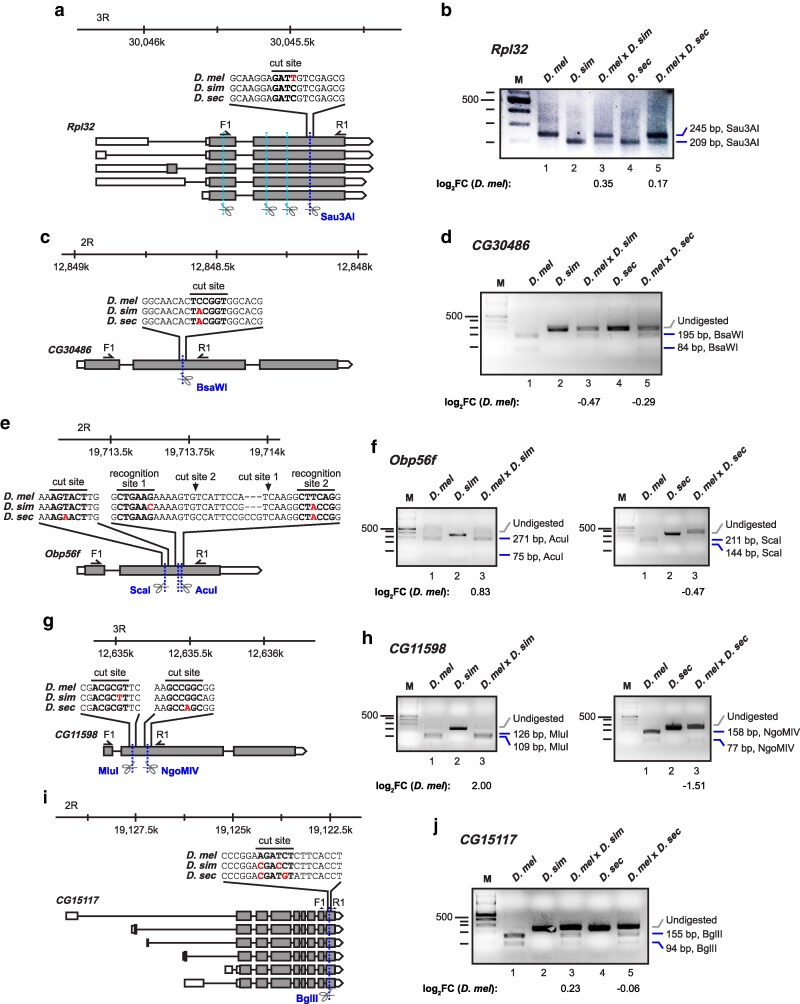
Comparison of transcript expression in *Drosophila* hybrids reveals divergent expression in a subset of Acps with fast evolving promoters. a–j) Schematic representation of the *Rpl32* (a), *CG30486* (c), *Obp56f* (e), *CG11598* (g), and *CG15117* (i) gene models and the strategy employed for analyzing differential transcript levels in *Drosophila* hybrids. RT PCRs from *D. melanogaster* × *D. simulans*, and *D. melanogaster* × *D. sechellia* hybrids were performed using primers flanking a restriction site with polymorphism(s) either *D. simulans* and/or *D. sechellia*. Primers are indicated as half arrows labelled F1 (forward) and R1 (reverse) and the restriction site alignment is indicated (bold) with polymorphisms in red. The restriction enzymes are also indicated with conserved cut/restriction sites in teal, and species-specific sites in blue. Cut/recognition sites are indicated in bold and polymorphisms in red. Representative agarose gels of species-specific restriction enzyme-digested RT PCRs for *Rpl32* (b) *CG30486* (d), *Obp56f* (f), *CG11598* (h), and *CG15117* (j). Experiments were performed for *D. melanogaster*, *D. simulans*, *D. melanogaster* × *D. simulans* hybrids, *D. sechellia*, and *D. melanogaster* × *D. sechellia* hybrids. Undigested (*D. simulans* and *D. sechellia*, grey) and digested (*D. melanogaster*, blue) bands are in indicated with sizes and the employed restriction enzymes. Differential gene expression is shown under each gel for the hybrid alleles as the log_2_ fold change for *D. melanogaster* vs either *D. simulans* or *D. sechellia*.

To determine expression levels of *CG30486*, *Obp56f*, and *CG15117* genes with high promoter evolution in hybrids from each allele, we identified single nucleotide polymorphisms (SNPs) that are part of restriction enzyme cut sites. We then designed an RT-PCR amplicon around these SNPs such that PCR fragments of different sizes for each species were obtained after a restriction digest and quantified the levels of PCR products in hybrids (Fig. 4, c to h). In addition, we analysed *CG11598* because its promoter contains a hotspot for sequence changes (Supplementary Fig. 1), while interspecies expression differences are moderate between the analysed species, and as a control for low promoter and expression changes, we used *Rpl32* (Fig. 4, a and b).

In this quantitative analysis of the two parent alleles in hybrids we expected that the changes in expression would reflect the changes in promoters. For *CG30486*, the *D. simulans* and *D. sechellia* alleles were dominant over the *D. melanogaster* allele (Fig. 4, c and d). Unexpectedly, our analysis of the fastest-evolving promoter genes, *Obp56f* and *CG11598*, revealed that the *D. melanogaster* allele was dominant in *D. melanogaster* × *D. simulans* hybrids, but that the *D. sechelia* allele was dominant in *D. melanogaster* × *D. sechelia* hybrids (Fig. 4, e to h). For *CG15117* and the control *RpL32*, no dominant allele is evident (Fig. 4, a, b, i and j).

**Fig. 5.**
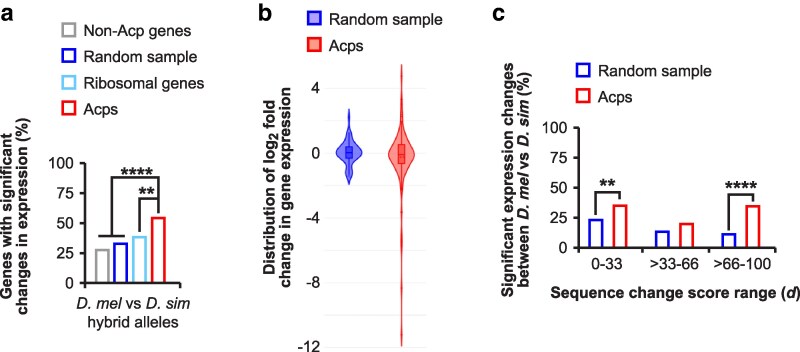
Expression of Acp genes diverges rapidly between *D. melanogaster* × *D. simulans* hybrids and correlates with mutation accumulation in promoters. a) The percentage of genes with significant changes in gene expression for Acps (red) compared to all analysed non-Acp genes (grey), a random sample of genes (blue), and ribosomal genes (light blue) calculated from differential gene expression analysis between *D. melanogaster*- and *D. simulans*-specific alleles in hybrid male accessory glands, excluding genes on the X chromosome. Statistically significant differences from FDR corrected non-parametric chi-squared tests are indicated by asterisks (**P ≤ 0.01, ****P ≤ 0.0001). b) Violin plots of log_2_ fold changes in gene expression distribution for the random genome sample and Acps calculated from differential gene expression analysis between *D. melanogaster*- and *D. simulans*-specific alleles in hybrid male accessory glands. c) Comparison of significant changes in log_2_ fold changes (>1 or <−1 log_2_ fold change) in gene expression in conservation promoter score (*d*) range 0 to 33%, >33 to 66%, and >66 to 100%. Expression changes for Acp (blue) and a random sample of genes were calculated from differential gene expression analysis between *D. melanogaster*- and *D. simulans*-specific alleles in hybrid male accessory glands. Statistically significant differences from non-parametric chi-squared tests are indicated by asterisks (**P ≤ 0.01, ****P ≤ 0.0001).

To expand this analysis to all Acp genes, we analysed RNA-seq data from *D. melanogaster* × *D. simulans* hybrids. In this analysis, Acp genes showed an increased frequency of expression divergence compared to all control groups (Fig. 5a). As seen with the initial species comparisons (Fig. 2b), fold change distribution was not significantly different between hybrid alleles (Fig. 5b). To check whether Acps with rapidly evolving promoters were enriched for significant expression changes between hybrid alleles, we separated genes into three conservation score groups based on *d* (0 to 33, >33 to 66, >66 to 100 *d*). Here, the percentage of genes with >1 or <−1 log_2_ fold changes were quantified for each group (Fig. 5c). Acps in the faster evolving group (>66 to 100 *d*) and slowest evolving group (0 to 33 *d*) were enriched for significant expression changes (Fig. 5c). Next, we analysed hybrid allele expression changes for the five genes tested in Fig. 4 (*Rpl32*, *CG30486*, *Obp56f*, *CG11598*, *and CG15117*). The direction of expression divergence was consistent for *Obp56f* and *CG15117*, but differed for the other genes likely reflecting the different *Drosophila* strains used (Canton S was used in this study, while the inbred line RAL-517 was used by Majane et al. (2024)) or technical sensitivity between methods (Table 1, Supplementary Dataset 1) (Majane et al. 2024).

**Table 1.** Differential log_2_ fold changes (log_2_FC) in gene expression for *D. melanogaster* versus *D. simulans* in non-hybrids, and for *D. melanogaster* versus *D. simulans* alleles in *D. melanogaster* × *D. simulans* hybrids. The final column shows the hybrid comparison values normalized to the non-hybrid values, calculated as (1/log_2_FC in non-hybrids) × log_2_FC in hybrids.

	log_2_ fold change in gene expression		log_2_ fold change in gene expression normalised to non-hybrids
Gene	*D. melanogaster* vs *D. simulans* in non-hybrids	*D. melanogaster* vs *D. simulans* alleles in *D. melanogaster* × *D. simulans* hybrids	Hybrids vs non-hybrids
*Rpl32*	0.56469732	−0.05277919	−0.09346456
*CG30486*	0.14792807	0.5723672	3.8692264
*Obp56f*	−1.35872671	−0.91834038	0.67588307
*CG11598*	−0.01453576	−0.25291426	17.3994466
*CG15117*	2.50180615	2.31701893	0.92613847

Taken together, our data show that promoter evolution drives changes in expression, because species-specific patterns of expression are either maintained in hybrids or dominated by one allele through *cis*- or *trans*-regulatory changes. Hence, our data implicate promoter evolution as a main source for fast changes in gene expression.

## Discussion

Among gene groups, Acp genes are among the fastest evolving genes when averaged. However, the protein coding regions of only a minority are under positive selection (Patlar et al. 2021). Here, we discover that the promoters of Acp genes are hotspots for sequence changes between species. We established a correlation between differences in gene expression among closely related *Drosophila* species and changes occurring in these promoters. Using RT-PCR combined with allele-specific restriction digests in *Drosophila* hybrids, we provide evidence that species-specific divergence in Acp expression is associated with rapidly evolving promoters. These findings underscore the critical role of promoter evolution in shaping the diversity of Acp expression across species. Numerous studies have shown that Sfp gene expression diverges rapidly between related species in both vertebrates and invertebrates (Coulthart and Singh 1988; Ellegren and Parsch 2007; Harrison et al. 2015; Kratochwil et al. 2018; Cridland et al. 2020; Nakadera et al. 2020; Flacchi et al. 2024). Moreover, such changes in expression can be accompanied by functional changes as shown for Sfps in *Lymnaea stagnalis* (Nakadera et al. 2020). Sfps were shown to have divergent gene expression profiles between *D. melanogaster* and *D. simulans* driven by a relaxation of selective pressures (Flacchi et al. 2024). However, Sfps with reproductive-tissue-specific expression were enriched for stabilizing and directional selection, while relaxed selection was the predominant mode of evolution among Sfp genes with other tissue-specific or non-tissue-specific expression (Flacchi et al. 2024).

Developmentally redundant Acps can likely tolerate drastic changes in gene expression since perturbation of processes such as sperm competition or the female postmating response does not influence fitness at the organismal level. Expression changes in Acps with postmating-specific roles could drive species divergence and fitness. Here, variability in Sfp concentrations could have drastic changes on reproductive success (Flacchi et al. 2024). For instance, Acps implicated in sperm competition experience high rates of duplication and loss, which could be attributed to increasing or decreasing their expression (Civetta and Ranz 2019). Promoter evolution likely has similar implications on expression, which could impact sperm competition. Modifications in the promoters of developmentally vital genes are probably constrained by compensatory mechanisms such as rapidly evolving enhancers or nested epistasis enhancer networks (Lin et al. 2022). Acps are unlikely restricted by strict stoichiometry from being part of enzymatic complexes, and this may explain their general expression divergence (Coulthart and Singh 1988; Ellegren and Parsch 2007; Harrison et al. 2015; Kratochwil et al. 2018; Cridland et al. 2020; Nakadera et al. 2020; Flacchi et al. 2024). As a consequence of changes in expression, however, binding properties likely change. To understand the impact of such changes in expression, we must first learn about the molecular functions of Acps in directing processes such as sperm competition (Lange et al. 2021; Rivard et al. 2021).

Of much interest is whether promoter evolution occurs at different rates and what the driving factors behind this are (Hill et al. 2021). Notably, it has been shown that promoters of genes required for suppression of transposon mobility evolve rapidly (Munafò et al. 2021; Nallasivan et al. 2021; McQuarrie et al. 2023, 2024). The accumulation of sequence changes in promoter regions has been associated with transposon mobility due to the presence of open chromatin around TSSs (Treiber and Waddell 2020). An example of how transposon driven promoter evolution can direct expression changes which affect biological processes is the outcome of *P*-element mutagenesis experiments observed in the *egghead* (*egh*) locus, which encodes an enzyme involved in glycosphingolipid biosynthesis (Soller et al. 2006). Following *P*-element mutagenesis, multiple base changes were detected in the first promoter region, leading to the emergence of the SP-insensitive allele, *egh^cm^*. Mutations in the *egh* gene give rise to pleiotropic phenotypes and the *egh^cm^* allele disrupts the neuronal connectivity essential for the female post-mating response and optic lobe development (Fan et al. 2005; Soller et al. 2006; Haussmann et al. 2013).

While testing interspecies expression divergence in hybrids, we expected species-specific expression levels to be maintained but instead observed allele-specific dominance in two Acps, where one allele consistently showed higher transcript abundance. A possible explanation for species-specific gene expression repression is a *cis* + *trans* interaction between diverged promoters and species-specific *trans*-acting factors in the hybrid background. Such interactions are supported by observations in *D. melanogaster* × *D. simulans* hybrids, where some Sfps often exhibit *cis* and *trans* regulation, indicating that multiple regulatory mechanisms likely contribute to Acp expression divergence (Majane et al. 2024). However, these may not show species-specific dominance as observed in two of the analysed Acps.

While our allele-specific RT-PCR combined with restriction digest provides valuable qualitative and quantitative insights into species-specific expression patterns, the resolution of standard agarose gel electrophoresis is limited. To support our quantifications, we analysed differential gene expression from RNA-seq of *D.* melanogaster × *D. simulans* hybrid male accessory glands. While some differences between the RT-PCR and RNA-seq results likely reflect variation in the *Drosophila* strains used and technical sensitivity, RT-PCR confirmed allele-specific expression patterns for a subset of genes, supporting the RNA-seq inference that hybrids exhibit allele-specific expression. This cross-validation supports the reliability of our findings despite methodological differences. To more accurately assess subtle expression differences between alleles, a denaturing gel analysis with radiolabelled or fluorescently labeled primers, as previously employed in high-resolution studies for *Dscam* isoform detection (Haussmann et al. 2019; Ustaoglu et al. 2019, 2024), would be required. In this study we analysed male whole-adult and hybrid male accessory gland RNA-seq data, though future analysezs of tissue-specific datasets could offer tissue-specific resolution without accounting for single-tissue expression.

In summary, our findings show that sequence changes in core promoters present between closely related species result in differences in gene expression. These changes can be maintained in hybrids of closely related species suggesting that promoter evolution dominates over the genomic background that alters cellular environment in hybrids. Moreover, since one allele can become dominant in hybrids, our results point out a prominent role for promoter changes in driving evolution.

## Supplementary Material

iyaf226_Supplementary_Data

## Data Availability

All data generated or analysed during this study are included in the supplementary information files. Supplementary information includes Supplementary Fig. 1 and Supplementary Dataset 1. Supplemental material available at GENETICS online.
