# Supplementary material for: Rapid promoter evolution of male accessory gland genes is accompanied by divergent expression in closely related Drosophila species
Source: Genetics. 2025 Oct 18;231(4):iyaf226. doi: 10.1093/genetics/iyaf226 (PMC12693585; doi:10.1093/genetics/iyaf226)

Supplementary Materials

Rapid promoter evolution of male accessory gland genes is accompanied by divergent expression in closely related *Drosophila* species

DAVID W. J. MCQUARRIE[1,2], FRANNIE H. S. STEPHENS[1], ALEXANDER D. FERGUSON[1], ROLAND ARNOLD[2,4,6], ALBERTO CIVETTA[3,6] AND MATTHIAS SOLLER[1,2,5,6]

Supplementary Fig. 1: Hot spots for sequence change accumulation in the Acp gene *CG11598* promoter

Plot of G scores between nucleotide changes and the differential accumulation of events along the fast-evolving *CG11598* promoter sequence. Sequences were aligned for *D. melanogaster*, *D. simulans*, *D. sechellia*, *D. yakuba*, and *D. erecta*. Positions in the alignment with significant stretches of substitutions (hot spots) are identified by black lines.

Supplementary Figure 1

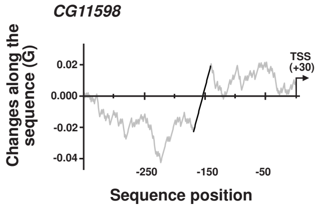

Supplement: iyaf226_Supplementary_Data [file iyaf226_supplementary_data.zip › Supplemental_Figure_1_GENETICS-2025-308664.pdf]
